# Natural Killer T Cells and Mucosal-Associated Invariant T Cells in Lung Infections

**DOI:** 10.3389/fimmu.2018.01750

**Published:** 2018-08-02

**Authors:** François Trottein, Christophe Paget

**Affiliations:** ^1^Univ. Lille, U1019 – UMR 8204 – CIIL – Centre d’Infection et d’Immunité de Lille, Lille, France; ^2^Centre National de la Recherche Scientifique, UMR 8204, Lille, France; ^3^Institut National de la Santé et de la Recherche Médicale U1019, Lille, France; ^4^Centre Hospitalier Universitaire de Lille, Lille, France; ^5^Institut Pasteur de Lille, Lille, France; ^6^Institut National de la Santé et de la Recherche Médicale U1100, Centre d’Etude des Pathologies Respiratoires (CEPR), Tours, France; ^7^Université de Tours, Tours, France

**Keywords:** natural killer T cells, mucosal-associated invariant T cells, mucosal immunity, infection, lung, bacteria, viruses, immunotherapy

## Abstract

The immune system has been traditionally divided into two arms called innate and adaptive immunity. Typically, innate immunity refers to rapid defense mechanisms that set in motion within minutes to hours following an insult. Conversely, the adaptive immune response emerges after several days and relies on the innate immune response for its initiation and subsequent outcome. However, the recent discovery of immune cells displaying merged properties indicates that this distinction is not mutually exclusive. These populations that span the innate-adaptive border of immunity comprise, among others, CD1d-restricted natural killer T cells and MR1-restricted mucosal-associated invariant T cells. These cells have the unique ability to swiftly activate in response to non-peptidic antigens through their T cell receptor and/or to activating cytokines in order to modulate many aspects of the immune response. Despite they recirculate all through the body *via* the bloodstream, these cells mainly establish residency at barrier sites including lungs. Here, we discuss the current knowledge into the biology of these cells during lung (viral and bacterial) infections including activation mechanisms and functions. We also discuss future strategies targeting these cell types to optimize immune responses against respiratory pathogens.

The respiratory tract constitutes one of the major sites of entry for pathogens. According to the vital nature of this organ, immune responses in the lung mucosa need to be finely regulated in a time- and strength-dependent manner. First, microbial-derived signals have to be rapidly integrated by the immune system in order to establish an efficient response that will allow containment and elimination of the pathogens. However, if not properly regulated, this inflammatory response can also turn into immunopathology leading to extensive tissue damages and organ failure with devastating outcomes for the host. Thus, the enrichment within the lung mucosa for cells endowed with potent immunoregulatory functions is paramount to combat infections as well as to maintain tissue functions and integrity. Among these cells, the family of unconventional αβ T lymphocytes recently emerged as central player in mucosal immunity.

These unconventional αβ T cells differ from conventional αβ T lymphocytes in many respects. After thymic egress, unconventional αβ T cells are readily capable of cytokine/chemokine secretion and cytolysis. Thus, unconventional αβ T cells occupy a unique niche in the immune system (spanning the innate-adaptive border of immunity). In contrast to its huge diversity in conventional T cells, the T cell receptor (TCR) repertoire of unconventional αβ T cells is fairly conserved and presents a limited number of V(D)J rearrangements. In addition, unlike conventional T cells that are restricted to the polymorphic major histocompatibility complex (MHC) class I and MHC class II molecules; unconventional αβ T cells recognize conserved antigens (Ags) [including (glyco)lipids, small metabolites, and modified-peptides] presented by the quasi-monomorphic MHC class Ib (e.g., HLA-E/Qa-1b, H2-M3) and MHC class-I like (e.g., group 1 and 2CD1, MR1) molecules.

Due to their quite low representation in rodents and the absence of specific tools to analyze them, unconventional αβ T cells have been overlooked by the scientific community for a long time. Thanks to the development of biological tools (e.g., gene-targeted mice and tetramers), the interest in understanding the functions and role of unconventional T cells in tissue homeostasis and diseases has tremendously grown over the last decades. As a result, the current literature quickly expands to the extent that unconventional T cells are now highly regarded by clinicians as attractive targets for future innovative cell-based immunotherapies. Here, we focused our attention on two major subsets of “innate-like” unconventional αβ T cells namely CD1d-restricted natural killer T (NKT) and MR1-restricted mucosal-associated invariant T (MAIT) cells. We discuss the recent advances in the functions of these cells during lung infections in mice and humans as well as the potential therapeutic opportunities based on harnessing the biology of these cells.

## General Features of CD1d- and MR1-Restricted T Cells

CD1d- and MR1-restricted T cells represent a heterogeneous population of cells (Table 1). In this section, we review the general features and biological tools available to analyze these particular subsets.

**Table 1:** Classification and characteristics of CD1d- and MR1-restricted T cells.

Family	Subsets	TCR repertoire	TCR ligands	Functions
CD1d-restricted T cells	Type I natural killer T (NKT) cells	Mouse: Jα14-Vα18 with Vβ8, Vβ7 and Vβ2 bias	Of either host or microbial origins	Cytokine secretion: NKT1: IFN-γ, IL-2, TNF-α; NKT2: IL-4, IL-9, IL-13; NKT17: IL-17, IL-21, IL-22; NKT10: IL-10; NKT_FH_-like: IL-5, IL-6, IL-21
		Human: Vα24-Jα18 with strong Vβ11 bias	α-GalCer α-GSL β-GSL α-GlcDAG	Cytotoxic capacity: perforin, granzyme

	Type II NKT cells	Mouse: diverse with oligoclonal Vα3.2-Jα9/Vβ8 and Vα8/Vβ8	Of either host or microbial origins	Cytokine secretion: T_H1_-related: IFN-γ, IL-2; T_H2_-related: IL-4, IL-13; T_H17_-related: IL-17; T_H0_-related: IL-10; T_FH_-related: IL-5, IL-6, IL-17
		Human: diverse?	Sulfatide, LPC, LPE, and PG	Cytotoxic capacity: perforin, granzyme

	Other CD1d-restricted cells	With αβ TCR	α-GalCer and microbial-derived lipids	Cytokine secretion: IFN-γ, IL-4, IL-13 and IL-17
		Mouse: Vα10-Jα50/Vβ8 (Type Ia NKT)		Cytotoxicity?
		Human: Vα24^−^/Vβ11^±^		
		With γδ TCR	m: Cardiolipin	Cytokine secretion: IFN-γ and IL-4
		Mouse: diverse including Vδ4^+^	h: α-GalCer and Sulfatide	Cytotoxicity?
		Human: Vδ1^+^		
		With δ/αβ TCR	α-GalCer and analogs	
		Vδ1^+^-Jα-Cα/Vβ		

Mr1-restricted T cells	MAIT cells	Mouse: Vα19-Jα33	Riboflavin derivatives (including uracils and lumazines)	Cytokine secretion: IFN-γ, TNF-α and IL-17A
		Human: Vα7.2-Jα33		Cytotoxic capacity

	Atypical Mr1-restricted T cells	Only reported in humans	Diverse metabolites? including pterins	Cytokine secretion: T_H1_-, T_H2_-, and T_H17_-related cytokines
				Highly variable between clones
		Diverse TCR repertoire		Cytotoxicity?

*α-GalCer, α-galactosylceramide; GSL, glycosphingolipid; α-GlcDAG, α-monoglucosyldiacylglycerol; LPC, lysophosphatidylcholine; LPE, lysophosphatidylethanolamine; PG, phosphatidylglycerol*.

### CD1d-Restricted T Cells

Natural killer T cells were the first member of the CD1d-restricted T cell family to be described. Originally, NKT cells were named after pioneer studies that observed the co-expression of—initially thought—NK cell-specific markers on a T cell subset (1, 2). Nowadays, this definition is obsolete since some NKT subsets do not necessarily express NK cell-related molecules (3, 4). By definition, CD1d-restricted T cells comprise the heterogeneous family of T cells that are restricted to the CD1d molecule [for reviews, see Ref. (5–7)]. Specifically, CD1d-restricted T cells can be further segregated into three groups called type I (or “invariant”) NKT, type II (or “variant”) NKT, and “others” CD1d-restricted T cells regarding their Ag specificity and TCR usage (8).

#### Type I NKT Cells

Type I NKT cells are the most characterized population of CD1d-restricted T cells. Type I NKT cells express a semi-invariant TCR composed of an invariant TCRα-chain (Vα14-Jα18 in mice and Vα24-Jα18 in humans) paired with a limited set of TCRβ-chains (5, 7). Type I NKT cells are also defined by their strong capacity to react through their TCR to the glycosphingolipid (GSL) alpha-galactosylceramide (α-GalCer) once inserted into the CD1d molecule (9). In addition, the type I NKT TCR has been shown to recognize a wide range of microbial-derived lipids (6, 10–12), which allows them to specifically respond in a broad set of infectious diseases. Of importance, type I NKT cells can also activate in response to a wide array of cytokines including IL-12, IL-1β, IL-18, IL-23, and IFN-β (13–15).

Type I NKT cells are relatively abundant in mice in which they populate both lymphoid and non-lymphoid tissues. On average, type I NKT cells represent 1–3% of the T cell pool and can account for up to 20–30% of total T cells in specific niches such as the liver (8). In sharp contrast, type I NKT cells are far less frequent in humans (100-fold lower frequencies at similar locations) at the exception of the omentum where they can represent 10–20% of total CD3^+^ cells (16). Of note, type I NKT cell subsets can be defined regarding the pattern of cytokines produced including NKT1 [IFN-γ-producing, pro-myelocytic leukemia zinc finger (PLZF)^low^/T-bet^+^], NKT2 (IL-4-producing, PLZF^high^/T-bet^−^), and NKT17 (IL-17-producing, RORγt^+^) subset (17, 18).

Upon activation, type I NKT cells can rapidly produce substantial amounts of multiple chemokines (including CCL2, CCL3, CCL4, CCL5, CCL11, and CXCL8) and cytokines such as TNF-α, IFN-γ, IL-4, IL-5, IL-10, IL-13, IL-17, IL-21, IL-22, and GM-CSF (19). This ability to produce a wide range of chemokines and cytokines (with sometimes opposite functions) depends on the nature of the stimuli (e.g., Ags and/or activating cytokines) as well as the cell subset triggered. Through this property, type I NKT cells have been shown to interact with as well as to influence the functions of numerous immune populations including neutrophils, dendritic cells (DCs), macrophages, NK cells, γδT cells, B and conventional T cells (20–28). In addition, type I NKT cells are capable to directly kill transformed and virally infected cells (28, 29).

#### Type II NKT Cells

Conversely, type II NKT cells represent a much broader family engulfing all other CD1d-restricted αβ T cells that react to lipids, but not to α-GalCer (8, 30, 31). Thus, type II NKT cells express a more diverse TCR repertoire. For instance, in mice, type II NKT cells use Vα1, Vα3.2, Vα4, Vα8, or Vα11 TCRα-chains paired with Vβ3, Vβ5, Vβ8, or Vβ11 TCRβ-chains and also comprise oligoclonal Vα3.2-Jα9/Vβ9 and Vα8/Vβ8 subsets (30). The TCR usage of type II NKT cells in humans is far less understood. However, type II NKT cells seem to be more abundant than type I NKT cells in humans (32). In line with their TCR diversity, type II NKT cells recognize Ags of various nature and origin (mammalian and microbial) including glycolipids (lyso)phospholipids and non-lipidic small molecules (33–38).

Due to the lack of specific tools to investigate their entire pool, the functions of type II NKT cells have mainly been proposed indirectly by comparing the phenotypes observed in Jα18- (which lack type INKT cells) versus CD1d-deficient (which lack both type I and type II NKT cells) mice in various settings. Despite being informative, this strategy is not without concerns for several reasons. First, this way of analysis imposes to speculate that NKT cell subsets do not regulate the functions of each other. A recent report also indicates that *Cd1d*^−/−^ mice present a significant enrichment for mature MAIT cells in the thymus and spleen that could account for the phenotype observed in these mice (39). In addition, the original *J*α*18*^−/−^ mouse line presented a severely impaired expression of many TRAJ segments (40). However, the recent generation of new mouse lines with specific deletion of TRAJ18 (41, 42) will allow to validate or not the pioneer experiments. Nevertheless, type II NKT cells appear to share conserved phenotypic and functional similarities with type I NKT cells including an effector memory phenotype, cytotoxicity, and secretion of numerous cytokines/chemokines (8, 31, 43, 44). Thus, they have been proposed to participate in antimicrobial responses, auto-immunity, and cancer (31, 43, 44).

#### Other CD1d-Restricted T Cells

Type I NKT and type II NKT cells account for the vast majority of the CD1d-restricted T cell pool. However, the recent literature indicates that the diversity of this family is much more complex than initially believed (45). For instance, the identification of a minute population of CD1d/α-GalCer tetramer-positive cells in *J*α*18*^−/−^ mice brought a new layer of complexity in the definition of NKT cells (46). These cells expressed a semi-invariant Vα10-Jα50 TCR with minimal conservation in the amino acid sequence of the TCRα chain expressed on type I Vα14-Jα18 NKT cells (46). Interestingly, α-GalCer-reactive non-type I Vα24^+^-Jα18^+^/Vβ11^+^ NKT cells can also be observed in humans (47). This includes Vα24^−^ Jα18^+^/Vβ11^+^ subset with public TCR repertoires and diverse populations of Vα24^−^ Jα18^−^/Vβ11^−^ with private repertoires (47). More surprisingly, some human γδT cell subsets can react to lipids (e.g., α-GalCer, sulfatide, andphosphatidylethanolamine) in the context of CD1d (48–51). Existence of CD1d-reactive γδT cells has also been proposed in mice (52). Last, an unusual population of T cells bearing a hybrid δ/αβ TCR was shown to recognize CD1d/α–GalCer complex (53). Further investigations are clearly required to evaluate the biological relevance of these rare subsets and to understand why they have been conserved across species. Altogether, this new literature highlights the huge—probably still expanding—diversity of the CD1d-restricted T cell family.

### MR1-Restricted T Cells

Mucosal-associated invariant T cells probably represent the main subset of T cells restricted to the MHC class I-related molecule, MR1 (54, 55). MAIT cells were initially named after their observation in the gut lamina propria of humans and mice (54). Despite their characteristics of tissue-resident memory T cells at barrier sites, MAIT cells can also populate lymphoid tissues and non-lymphoid organs (8) such as the liver (56, 57). In addition, MAIT cells circulate through the bloodstream where they can represent up to 10% of the human T cell compartment. On the contrary, MAIT cells are relatively rare in standard laboratory mouse strains (55, 58).

Mucosal-associated invariant T cells are characterized by the expression of a semi-invariant TCR composed of a canonical TCRα chain (Vα19-Jα33 in mice and Vα7.2-Jα33 in humans) associated with a restricted set of Vβ segments (54, 55, 58, 59). Several subsets of human and mouse MAIT cells have recently been identified, including MAIT cell precursors (39, 60) and non-MAIT mature human MR1-restricted T cells using diverse αβTCR (61–63) that may have specific functions such as tumor Ag recognition.

Through their TCR, MAIT cells recognize small intermediate metabolites from the riboflavin (vitamin B2) pathway of bacteria (64). The first defined Ags were microbial pterin-like compounds presenting a ribityl moiety (64). In addition, the non-enzymatic reaction between a riboflavin precursor(5-amino-6-d-ribitylaminouracil) and small aldehydes (glyoxal/methylglyoxal) of both microbial and host origin generates two instable forms namely 5-(2-oxoethylideneamino)-6-d-ribitylaminouracil and 5-(2-oxopropylideneamino)-6-d-ribitylaminouracil (65). These two molecules represent potent Ags that are able to bind into MR1 and activate mouse and human MAIT cells (65, 66).

These discoveries subsequently enabled the development of mouse and human MR1 tetramers loaded with MAIT cell Ags that represented a major breakthrough in the investigation of MAIT cell biology including development, phenotype, and functions (39, 58, 67). Reminiscent with NKT cell biology, MAIT cells can respond to TCR signals and/or to various activating cytokines including IL-12, IL-1β, IL-18, and IL-23 (56, 68, 69). Upon activation, MAIT cells produce large amounts of Th1- and Th17-related cytokines such as IFN-γ, TNF-α, IL-17A, and IL-22 (70). Additionally, MAIT cells are capable of efficiently kill bacteria-infected cells (71, 72).

Due to their scarce representation in common laboratory mouse strains, understanding MAIT cell biology is challenging by comparing wild-type (WT) and *Mr1*^−/−^ mice. To better assess their functions, transgenic mice (*V*α*19i*Tg × Cα^−/−^) displaying high content of MAIT cells have been developed (73). However, the use of these mice is not without concerns since MR1 tetramer-positive cells can still be detected in Vα19i TCR transgenic (Tg) mice that lack MR1 (58). In addition, a certain abnormality in MAIT cells have been reported in these Tg mice (67). Nevertheless, MAIT cells can be found at high frequencies in unusual inbred mouse strains such as *Mus musculus castaneus* (CAST mice) (74). Interestingly, the reason for this MAIT phenotype in CAST mice relies on a single locus located on chromosome 14. Thus, a congenic mouse presenting a high level of MAIT cells (20× compared to classical C57BL/6) on a C57BL/6 background (named B6-MAIT^CAST^) was generated (74) and will be likely to be helpful to investigate the functions of MAIT cells.

Given their cytokine profile and cytotoxic capacity, MAIT cells intuitively emerged as cell subsets specialized in host defense against bacteria. However, recent evidences indicate that MAIT cells get activated in many pathological situations such as acute and chronic viral infections (68, 75–78), solid cancers and hematological malignancies (79–82), as well as many inflammatory disorders including type I and type II diabetes (83, 84), inflammatory bowel disease (85, 86), graft-versus-host disease (87), chronic obstructive pulmonary disease (88, 89), and multiple sclerosis (90, 91).

## Lung CD1d-Restricted NKT Cells and MR1-Restricted Mait Cells in Health

### CD1d-Restricted NKT Cells

In mice, type I NKT cells account for around 2–5% of lung-resident T lymphocytes. Lung type I NKT cells are mainly resident either as marginated cells within the vasculature or located in the lung interstitial parenchyma (92, 93). The lungs are particularly enriched for NKT17 cells compared to the vast majority of tissues (3). Interestingly, type I NKT cell location in the lung tissue is strongly dependent on the subsets. While NKT1 and NKT2 cells are predominantly found in the vasculature, NKT17 cells are at frontline within the lung parenchyma (93, 94). However, the factors that regulate their homing and homeostasis in the lung tissue are yet to be defined. Of note, microbiota seems to regulate lung type I NKT cell homeostasis since germ-free mice display an increase frequency of type I NKT cells, which is dependent on hypermethylation and increase levels of CXCL16 (95).

### MR1-Restricted MAIT Cells

Mucosal-associated invariant T cells are also present in the lung tissue of mice in which they account for approximately 2 and 0.3% of resident T lymphocytes in C57Bl/6J and BALB/c, respectively (67). Akin to NKT cells, lung MAIT cells predominantly display a phenotype of IL-17-producing cells defined by high expression of IL-7Rα and IL-18R1 and the lack of NK1.1 expression (67). In line, stimulation of purified lung MAIT cells of naive mice induced strong IL-17A production but little IFN-γ (67). In addition, they present a phenotype of effector memory cells (CD44^high^ CD62L^low^). The precise pulmonary niches of MAIT cells have not been determined, so far, but should be revealed soon, for instance, using antibody (Ab)-mediated *in vivo* labeling. How lung MAIT cells rely on commensal bacteria is currently unknown; however, there is a severe impairment in MAIT cells in the thymus, spleen, and gut of germ-free mice (39, 54). While NKT cells and MAIT cells appear to patrol the lungs in the steady state, their contribution to lung physiology and tissue integrity remains to be determined.

## CD1d-Restricted NKT Cells and MR1-Restricted Mait Cells in Lung Infections

A large body of evidence in both preclinical and clinical settings has recently suggested a key role for both NKT cells and MAIT cells in host response against lung pathogens (Table 2). Here, we compared the mode of activation and functions of NKT cells and MAIT cells in infectious respiratory diseases. We focused our interest on pathogens that provide data for both populations.

**Table 2:** *In vivo* role of natural killer T (NKT) cells and mucosal-associated invariant T (MAIT) cells in lung infections.

Microorganisms	Subsets	Cytokine produced	Activation mechanisms	Functions	Phenotype in mouse lines	Reference
*Mycobacterial infections*	Type I NKT	IFN-γ	CD1d- and cytokine-mediated (IL-12 and IL-18)	Not essential for host defense	No differences in survival or bacterial loads using *J*α*18*^−/−^ and/or *CD1d*^−/−^ mice	(96–106)
				–		
				Control granuloma formation (controversial)		

	MAIT	IFN-γ	–	Early but not late control of bacterial outgrowth	Increased bacterial burden in *V*α*19i* × *Mr1*^−/−^ compared to *V*α*19i* × *Mr1*^+/+^ mice	(69, 107)
		IL-17A (indirect evidences)			–	
					Increased bacterial loads in *Mr1*^−/−^ mice compared to wild-type (WT) (only observed at early stage)	

*Francisella. tularensis (LVS strain)*	Type I and type II NKT	IFN-γ	CD1d-dependent (ligand yet to be defined)	Immunoregulatory functions that prevent secretion of pro-inflammatory cytokines and recruitment of inflammatory cells	Enhanced mortality and weight loss in *J*α*18*^−/−^ and Vα14i Tg mice	(108)
			Indirectly evidenced analyzing Nur77 expression	–	–	
				Opposite functions for type I and type II NKT cells to be considered	Protection in *CD1d*^−/−^ mice	

	MAIT	IFN-γ	MR1- and cytokine-mediated (IL-12 and IL-18)	Recruitment of adaptive CD4^+^ and CD8^+^ T cells through reprogramming of monocytes int moDC	Higher bacterial burden in *Mr1*^−/−^ mice	(109, 110)
		TNF-α				
		IL-17	Only demonstrated *in vitro*			
		GM-CSF				

*Streptoccocus pneumoniae (serotype 1 and 3)*	Type I NKT	IFN-γ	Dependent on the strain used: Serotype 3: CD1d-mediated (α-glucosyldiacylglycerol from the pathogen) and cytokine-mediated (IL-12)	Neutrophilia	Enhanced mortality, weight loss and bacterial loads in *J*α*18*^−/−^ mice compared to WT controls	(92, 111–115)
		IL-17A	Serotype 1: cytokine-mediated: IL-12 for IFN-γ	–		
				Bacterial clearance		

	MAIT	IFN-γ	MR1-dependent	Cytotoxic activity *in vitro* on *Sp*-infected airway epithelial cells	To be tested	(116)
		IL-17A	Only tested *in vitro*	–		
				Killing efficacy is isolate specific and dependent on riboflavin biosynthesis pathway activity		

*Infuenza A virus (H1N1, H3N2, and H3N1)*	Type I (and II) NKT	IFN-γ	*H1N1* (IFN-γ): CD1d-dependent	*Pro-inflammatory functions*: promote Influenza A virus (IAV)-specific CD8+ T cell response by: (1) inhibition of MDSC functions (2) Regulation of DC migration to the lung-draining lymph nodes Regulatory functions: (1) Control of inflammatory monocyte recruitment (2) Control of IFN-γ production by NK cells	Increased mortality and weight loss in *J*α*18*^−/−^ and *Cd1d*^−/−^mice	(117–120)
		IL-4/IL-13	*–*		–	
		IL-22	*H3N2* (IL-22): CD1d-independent and cytokine-mediated: IL-1β and IL-23 through engagement of TLRs and RNA helicases		Enhanced tissue damages in *J*α*18*^−/−^ mice	
					–	
			*–*		Delayed viral clearance in *J*α*18*^−/−^ and *Cd1d*^−/−^ mice (H1N1)	
			*H3N1* (IL-4/IL-13): TLR7-dependent		No changes observed in *J*α*18*^−/−^ mice (H3N2)	

	MAIT (only tested with human MAIT cells)	IFN-γ	MR1-independent	Cytotoxic activity on IAV-infected cells	To be done	(68, 75)

		TNF-α	Cytokine-driven: IL-12, IL-18, and type I IFNs			

### Mycobacteria

Mycobacteria are the causative agents of tuberculosis (TB) due to a group of closely related species including *Mycobacterium tuberculosis, Mycobacterium bovis, M. microti*, and *M. africanum*. More than a billion of people have died of TB during the last three centuries. Nowadays, TB remains the major cause of death due to infection, causing an estimation of 10 million new cases and ~2 millions of fatalities per year worldwide (121). In addition, one-quarter to one-third of the global human population is latently infected (122, 123), constituting a large reservoir for future spread of active TB. TB control mainly relies on a 100-year-old vaccine [Bacillus Calmette–Guérin (BCG)] (124) that saved the lives of many children without impacting on TB prevalence. In addition, the emergence of resistances for antituberculosis drugs, developed several decades ago, becomes worrying (125).

#### NKT Cells

The role of NKT cells during *Mycobacterium* infection has been extensively studied in preclinical models (96–98, 126). Early type I NKT cell activation (IFN-γ release and cytotoxicity) has been reported during *M. bovis* BCG and *M. tuberculosis* infections (96, 99, 126). In addition, type I NKT cells can also exert antimycobacterial effector function *in vitro* through the secretion of GM-CSF (127). The mechanisms that drive type I NKT cell activation have been shown to rely on both adaptive and innate cues such as CD1d-mediated Ag recognition and the activating cytokines IL-12 and IL-18 (97, 126). Several mycobacterial-derived lipids such as phosphatidylglycerol, cardiolipin, or phosphatidylinositol activate type II but not type I NKT cells (35) even if an early study has suggested CD1d-dependent antigenic properties for phosphatidylinositol mannoside on type I NKT cells (128). Despite their early activation during mycobacterial infections, type I NKT cells do not appear to have a critical role in the control of TB (100–104) despite some controversial results have been reported regarding granuloma formation (96, 105, 126). Akin to *J*α*18*^−/−^ mice, CD1d-deficient mice did not significantly differ in their susceptibility to mycobacterial infection compared to control mice (106). This suggests that neither type I nor type II NKT cells play a cardinal role in TB outcome in mice.

Several clinical reports indicated that patients with active TB present reduced levels of circulating NKT cells compared to patients with latent forms (129–134). However, NKT cells from patients with active TB present an activated phenotype and can secrete high amounts of IFN-γ (129, 135). Of note, NKT cell activation in TB patients leads to PD-1 expression at cell surface and subsequent apoptosis of IFN-γ-producing NKT cells, a phenomenon that can be reversed through PD-1 blockade (136, 137). Human NKT cells have also been shown to exert direct bactericidal effects on *M. tuberculosis*-infected macrophages (126). In addition, some NKT cells from pleural effusion of TB patients present characteristics of T_FH_-like cells (138). Thus, it is possible that T_FH_-like NKT cells produce IL-21 to participate in local B cell response against *M. tuberculosis* (138).

Although preclinical models do not point toward a key role for NKT cells in TB, their rapid response and immunoregulatory functions poised them as an interesting target for cell-based therapies. In line, exogenous activation of type I NKT cells with α-GalCer (alone or in combination with classical chemotherapy) protected mice from lethal TB (96, 99, 139, 140). The molecular determinants that drive this protective effect are not fully understood but are likely to rely on enhanced innate response such as IFN-γ production and cytotoxicity. Moreover, α-GalCer (or analogs) incorporation into BCG vaccine enhances immune responses in mice by modulating T cell priming *via* type I NKT cell activation (141). α-GalCer can also exert adjuvant effect on TB vaccines using the sublingual route by enhancing Ag-specific IFN-γ-producing T cells (142). In addition to its primary application as a vaccine for TB prevention, epidemiological studies have suggested that BCG vaccination may prevent IgE-mediated allergic diseases in mice and humans (143, 144). Interestingly, the BCG-mediated IgE response suppression appears to be dependent on IL-21 secretion by type I NKT cells that mediates specific apoptosis of IgE-expressing B cells (145).

#### MAIT Cells

The role of MAIT cells in preclinical model of TB has been explored. Mouse MAIT cells can inhibit intracellular growth of *M. bovis* BCG in macrophages (107). This effect relies on MR1- and IL-12-dependent IFN-γ secretion by MAIT cells (107). Interestingly, upon BCG infection at low dose, *Mr1*^−/−^ mice displayed a higher bacterial burden in the lung during the early course of infection compared to MAIT cell-proficient mice. However, similar bacterial loads were observed at later stage of infection (107) suggesting an important role for MAIT cells in early control of bacterial growth although inefficient to prevent long-lasting establishment of the bacteria in the lung tissue. The role of MAIT cells in controlling mycobacterial infection has also been evaluated using *V*α*19i* Tg mice (69). Upon intravenous injection of *M. abscessus, V*α*19i* Tg × *Mr1*^−/−^ mice displayed slightly but significant higher bacterial loads than their *Mr1*^+/+^ littermates (69). Despite having an increased proportion of MAIT cells, it is important to mention that *V*α*19i* Tg × *Mr1*^+/+^ mice have a comparable bacterial burden compared to WT mice (69). Thus, MAIT cells play a limited protective role in the early course of mycobacterial infections.

Akin to NKT cells, clinical studies in patients with active TB indicated a striking decrease in frequency of circulating MAIT cells compared to healthy controls (69, 71, 146). This strong reduction is paralleled with an enrichment of MAIT cells in the lung tissue and pleural effusions of TB patients (71) suggesting a recruitment to the inflamed tissue. In addition, one can argue that this relative disappearance can also rely on other mechanisms such as increased apoptosis (147) or TCR downmodulation (rendering them virtually undetectable) as a consequence of overactivation. Indeed, MAIT cells from blood and pleural effusions of TB patients displayed a phenotype of activated/memory cells (CD69^+^ CD45RO^+^) associated with a higher capacity to produce antimycobacterial cytokines such as IFN-γ and TNF-α (147). This phenotype can be further exemplified since MAIT cells from these patients had a reduced response to unspecific restimulation (147). Reminiscent with the data generated in studies on NKT cells, MAIT cells from TB patients expressed high levels of PD-1 and *ex vivo* PD-1 blockade restored IFN-γ production by these latter (147). Human MAIT cells can also mediate cytotoxicity against mycobacteria-infected cells. Thus, they can recognize and kill *M. tuberculosis*-infected cells including DCs and lung epithelial cells in a MR1-dependent manner (71, 148, 149). Of note, non-MAIT Vα7.2-Jα12 MR1-restricted T cells can also efficiently kill BCG-infected THP-1 (a monocytic cell line) (72).

### Other Bacteria

Concurrent literature for NKT cells and MAIT cells in the context of bacterial lung infection is rather limited. Some data are nevertheless available for models of pneumonia induced by the Gram-negative bacterium *Francisella tularensis* or by the Gram-positive bacterium *Streptococcus pneumoniae*.

#### *Francisella* *tularensis*

*Francisella tularensis* is the causative agent of tularemia (also referred to as rabbit fever); a zoonosis that spreads *via* arthropod vectors after contact with infected animals (150). Depending on the site of infection, tularemia can lead to several clinical manifestations including pneumonia, the most deadly form of the disease with a mortality rate up to 60% (151). Incidence rates have drastically dropped over the last century despite some local outbreaks have been noticed over the last decades (152). In addition, *F. tularensis* can be considered as a potential agent for bioterrorism (153). Given the high virulence of *F. tularensis*, preclinical data mainly derived from attenuated strains such as *F. tularensis* Live Vaccine Strain (LVS).

#### NKT Cells

Surprisingly, the role of NKT cells has only recently emerged in experimental pulmonary tularemia (108), a model of immunopathology that can be related to sepsis-like disorder (154). To explore this, authors studied survival and body weight loss in various mouse strains including *J*α18^−/−^, *Cd1d*^−/−^, and *V*α*14i* Tg mice. Unfortunately, due to the apparent variability in this model and the lack of data using all mouse lines in a single experiment, interpretation of the results is not straightforward (108). Thus, *J*α*18*^−/−^ and *V*α*14i* Tg mice were more susceptible to infection compared to WT controls while *Cd1d*^−/−^ mice were more resistant. Altogether, these data implied that NKT cells probably play deleterious role during pulmonary tularemia even if data obtained with *J*α*18*^−/−^ mice do not support this hypothesis. To explain this, authors claimed that CD1d-deficient mice represent a better model than *J*α*18*^−/−^ mice to study the role of NKT cells in disease (108). However, several additional hypotheses are worth mentioning to explain these results. First, it is possible that type I NKT and type II NKT cells play opposite functions during pulmonary tularemia. Although, *V*α*14i* Tg mice present a similar phenotype than *J*α*18*^−/−^ mice, it has to be kept in mind that type I NKT cells generated from *V*α*14i* Tg mice present unique characteristics compared to *bona fide* type I NKT cells and a profound bias in their CD8^+^ T cell repertoire (155). Given the role of MAIT cells during pulmonary tularemia (see below), the phenotype observed in *Cd1d*^−/−^ mice can also at least partially rely on the high level of MAIT cells in these mice (39). Conversely, *J*α*18*^−/−^ mice are likely to have reduced MAIT cell frequencies since 60% of the TCRα repertoire diversity is actually lacking in these mice (40).

The resistance of *Cd1d*^−/−^ mice to tularemia is mainly associated with enhanced formation of tertiary lymphoid structures in lungs of infected mice associated with mitigated inflammation including reduced pro-inflammatory cytokines and inflammatory cell recruitment (108). Collectively, NKT cells have deleterious functions during tularemia although a more refine analysis of the potential differential roles of type I NKT and type II NKT cells in this model should be considered.

#### MAIT Cells

CD4^−^CD8^−^ double-negative (DN) T cells have been shown to represent a major responding T cell subset in the lungs of LVS-infected mice (156). Despite the lack of specific tools, authors unequivocally demonstrated a couple of years later that this strong accumulation of DN T cells during pulmonary LVS infection was mainly attributable to MAIT cells (109). Indeed, most of these cells expressed transcripts for the invariant Vα19-Jα33 α-chain rearrangement associated with TCR β-chains containing Vβ6 or Vβ8 segments (109). In addition, the accumulation of DN T cells was absent in *Mr1*^−/−^ mice. Interestingly, *Mr1*^−/−^ mice exhibited higher bacterial burden in the lungs compared to WT mice from day 10 postinfection (109).

During *in vivo* infection, MAIT cells produced antibacterial cytokines including IFN-γ, TNF-α, GM-CSF, and IL-17A and contributed to recruitment of adaptive CD4^+^ and CD8^+^ T cells (109, 110). Interestingly, MAIT cells controlled the recruitment of activated CD4^+^ T cells through GM-CSF-mediated reprogramming of monocytes into monocyte-derived DCs (110).

In addition, cultures of MAIT cells with LVS-infected monocytes led to a MAIT cell-dependent reduction in bacterial growth, a mechanism that relied on secretion of IFN-γ, TNF-α, and nitric oxide (109). *In vitro* activation of MAIT cells in this model was dependent on MR1 and the activating cytokines IL-12 and IL-18 according to the strain of bacterium used (109, 157). Altogether, MAIT cells appear as important antibacterial players during tularemia.

#### *Streptococcus* *pneumoniae*

*Streptococcus pneumoniae* (the pneumococcus) is the major bacterium responsible for community-acquired pneumonia in western countries and accounts for ~2 million of deaths per year worldwide. In normal conditions, *S. pneumoniae* colonizes asymptomatically nasopharynx of healthy individuals. However, when the immune equilibrium is broken, pneumococcus carriage can lead to mild disease such as otitis media or sinusitis and more occasionally turns into severe complications such as pneumonia, sepsis, and meningitis (158). In addition, presence of *S. pneumoniae* is often found in biological fluids of hospitalized patients for severe influenza A infection (159) as well as patients with exacerbated chronic lung inflammation such as chronic obstructive pulmonary disease (160).

Despite vaccination is an efficient strategy to prevent pneumococcus spread and to control infections, the available vaccines have, however, some issues [for reviews, see Ref. (161, 162)]. In addition, the emergence of antibiotic-resistant strains (163) represents an important threat for the management of pneumococcal infections in clinics.

#### NKT Cells

Many studies have highlighted an important role for type I NKT cells in host response against pulmonary pneumococcal infection. Using *S. pneumoniae* serotype 1 or 3 strains, we and others reported that *J*α*18*^−/−^ mice were more susceptible to infection and displayed higher bacterial burden compared to WT controls (111–113). The underlying mechanisms of this protective activity rely on IFN-γ secretion by type I NKT cells that regulate early recruitment of neutrophils (113). Elaborately, type I NKT cell-derived IFN-γ controls the secretion of CXCL2 and TNF-α (113), two critical mediators of lung neutrophilia. Type I NKT cells migrate to the lung parenchyma only 24 h after *S. pneumoniae* infection in a CCL17-dependent manner, a mechanism, which is crucial for mouse survival (92). The mechanisms involved in type I NKT cell activation during *S. pneumoniae* infection have been extensively explored and depends on activating cytokines, pneumococcal-derived Ag, or both according to the strain studied. First, akin to other bacteria, the cell wall of several *S. pneumoniae* strains is constituted of glycolipids (α-glucosyldiacylglycerol) that can serve as CD1d-restricted type I NKT cell Ags (114). The recognition of this Ag *in vivo* is important for host response to *S. pneumoniae* (114). The general mechanism that allows accessibility and loading of such microbial-derived Ags into CD1d are currently unknown and deserve further investigations. In addition, type I NKT cell activation during pneumococcal infection is also dependent on the release of IL-12 especially by CD103^+^ DCs (112, 115). No data are currently available regarding type II NKT cells during pneumococcal infection.

Exogenous activation of type I NKT cells protects against lethal *S. pneumoniae* infection independently of the strain used (113, 164). Mechanistically, this protective activity relied on IFN-γ and IL-17A production and subsequent neutrophil recruitment that controlled bacterial elimination (164). While these protective effects have been obtained using α-GalCer, exogenous activation of type I NKT cells with α-mannosylglycolipids has also been shown to partially protect mice against pneumococcal infection (165).

Surprisingly, despite strong evidence for a critical role in host response to pneumococcus in experimental models, no clinical studies have so far addressed the dynamics and functions of NKT cells in patients with severe *S. pneumoniae*-driven pneumonia.

Type I NKT cells have been shown to provide help for B cells in mounting Ab responses (26, 166). In line with this literature, NKT cells have important role in the production of specific antipneumococcal Abs and class switching in response to pneumococcal vaccines in experimental models (167, 168). A recent clinical study enrolling vaccinated subjects with the 23-valent pneumococcal polysaccharide vaccine reported a positive correlation between frequency of circulating type I NKT cells and the serum concentrations of specific IgG directed against serotypes 14 and 19F (167). Interestingly, injection of liposomes containing synthetic NKT cell Ags and pneumococcal capsular polysaccharides led to the generation of long-term memory B cell response (169, 170). Generation of such a response was dependent on the recognition of lipids and capsular polysaccharide antigens by type I NKT cells and B cells, respectively, to elicit cognate and direct NKT–B cell interactions. Thus, harnessing NKT cell functions (adjuvant effect or B cell help) during pneumococcal vaccination might represent an interesting avenue of research in the future.

Of note, pneumococcal infection and vaccination have been proposed to reduce airways allergic diseases as a result of immune modulation in both experimental and clinical studies (171–173). Interestingly, this mechanism relied on the regulatory functions of some components of the pneumococcus including the bacterial toxin pneumolysin and type-3-polysaccharide (174). These molecules induced the generation of regulatory T cells that control type I NKT cell accumulation and limit the development of airway hyperresponsiveness (174).

#### MAIT Cells

Early works suggested that MAIT cells do not react to *Streptococcus* group A (69). However, genomic and transcriptomic analyses of numerous strains indicate that *S. pneumoniae* expresses enzymes involved in the synthesis of riboflavin metabolites (175, 176) as well as a highly conserved riboflavin operon (177). Thus, activation and functions of MAIT cells during pneumococcal infection have recently been investigated. Human MAIT cells can produce IFN-γ in response to accessory cells infected by *S. pneumoniae* clinical isolates (116, 177). Interestingly, the magnitude of MAIT cell response differed from one isolate to another and was proposed to depend on genetic variations in their expression of the riboflavin pathway (116) suggesting a role for MR1-dependent Ags in this model. Conversely, another study suggested that MAIT cell activation, in response to THP-1 cells cultured with fixed pneumococci was unchanged upon MR1 blockade but was fully abrogated following IL-12 and IL-18 neutralization (177). This discrepancy might stem from differences in experimental design as well as in the level of metabolic activity (riboflavin metabolism) of the various strain used. In line with the first hypothesis, the use of macrophages in the culture assay led to IFN-γ release by MAIT cells in a MR1-dependent manner (177). Environmental factors can also determine the generation of MAIT cell Ags by the pneumococci. Thus, modulation in riboflavin availability in the culture assay significantly influenced MAIT cell activation in response to pneumococcus-infected DCs. Specifically, excess of riboflavin blunted MAIT cell response while culture in riboflavin-free assay medium increased MAIT cell activity (116, 177). In addition, heat stress can induce the riboflavin operon in *S. pneumoniae* resulting in higher MAIT cell Ag availability (177).

Using *in vivo* models of pneumococcal infections with virulent or avirulent strains in Vα19i Tg × Cα^−/−^ mice, a small proportion of lung MAIT cells produced IFN-γ and IL-17A (116). Although a more detailed kinetic analysis is required, these levels were relatively low compared to cytokine production observed for other unconventional T cell subsets in this model including γδT cells and NKT cells (112, 178, 179). However, the precise functions of MAIT cells in pneumococcus-induced pneumonia are currently unknown since data from either *Mr1*^−/−^ or Vα19iTgCα^−/−^*Mr1*^−/−^ mice are not yet available.

Akin to NKT cells, the phenotype and dynamics of MAIT cells in patients with severe pneumonia associated with presence of pneumococci are rather limited. Of note, the levels of circulating MAIT cells in critically ill patients with severe bacterial infections were markedly decreased compared to age-matched healthy donors (180). Surprisingly, the decrease in MAIT cell frequency was less pronounced in patients with streptococcal infections including pneumonia compared to non-streptococcal infections (180). This might imply a minimal role for MAIT cells during *S. pneumoniae*-induced pneumonia in humans although this observation should be confirmed by the use of MR1 tetramers.

### Influenza A Virus (IAV)

Influenza A viruses belong to the family of *Orthomyxoviridae* viruses and are characterized by a segmented single-stranded RNA genome of negative polarity. IAV can be further segregated regarding the expression of two important proteins namely hemagglutinin and neuraminidase proteins. IAV are responsible for seasonal highly contagious infections characterized by a severe pulmonary immune pathology leading to human morbidity and mortality (181). About five millions of clinical cases and 250,000 to 500,000 deaths are reported worldwide every year (182, 183). More sporadically, transversal infections of animal strains to humans or co-infection with multiple IAV strains in individuals can result in re-assortment of genes generating highly virulent new strains leading to life-threatening pandemics (184, 185). Recent epidemiological studies also indicated that hospitalized patients for severe pneumonia during IAV epidemics or pandemics are often co-infected with bacteria including *S. pneumoniae* (methicillin-resistant) *Staphylococcus aureus, Pseudomonas aeruginosa*, and *Haemophilus influenza* (186). These secondary bacterial infections contribute significantly to the excess morbidity and mortality of influenza [for a review, see Ref. (186)]. Mechanistically, infection with IAV dampens innate antibacterial immunity and alters pulmonary barrier functions thus favoring local bacterial outgrowth and dissemination from the lungs [for reviews, see Ref. (187, 188)]. Considering the clinical impact of bacterial superinfection post-influenza, the development of preclinical models should be encouraged for proper translational studies.

#### NKT Cells

We and others have demonstrated the pivotal role of type I NKT cells in host response to IAV infection. Keeping in mind that they represent a heterogeneous population composed of subsets endowed with specialized functions, type I NKT cells have been shown to exert multiple functions during the course of IAV infection. Despite mechanisms differ from one experimental setting to another, type I NKT cell deficiency has been consensually shown to increase susceptibility (survival, enhanced inflammation) in several experimental influenza (H1N1, H3N1, and H3N2) infection models (117–119). For instance, a pioneer study described a new mechanism in which type I NKT cells could inhibit the suppressive functions of myeloid-derived suppressor cells resulting in enhanced IAV-specific CD8^+^ T cell response, enhanced viral clearance, and increased survival (119). We also described a role for type I NKT cells in mounting IAV-specific CD8^+^ T cell response (118). In our model, this effect relied on NKT cell-mediated control of lung CD103^+^ DC emigration to the draining lymph nodes (118). In parallel, type I NKT cells exert anti-inflammatory functions during IAV infection, thus preventing immunopathogenesis. First, type I NKT cells control the recruitment of inflammatory monocytes (117) as well as the production of IFN-γ by NK cells (118), two mechanisms that can blunt IAV-associated pathogenesis (189, 190). In addition, type I NKT cells produced the tissue protective cytokine IL-22 during the early course of IAV infection (120). Although it did not affect viral loads *in vivo* (191), IL-22 protected against epithelial damages due to viral replication (120, 192). Through this mechanism, IL-22 might reduce secondary bacterial infection post-influenza (191, 193).

The activation mechanisms of type I NKT cells during IAV infection have been proposed to rely on either CD1d-dependent or CD1d-independent pathways. First, type I NKT cells have been shown to directly control the suppressive activities of myeloid-derived suppressor cells in a CD1d-dependent manner (119). The nature of the ligand involved is yet to be defined but appears to be a neo-synthesized GSL of host origin (119). On the other hand, we demonstrated that IL-22 production by type I NKT cells during IAV infection was dependent on IL-1β and IL-23 secretion by accessory cells and did not require CD1d (120).

The potential role of type II NKT cells during IAV is currently unknown. Of note, *CD1d*^−/−^ mice are more susceptible to H1N1 IAV infection than *J*α*18*^−/−^ mice although no differences between genotypes were observed regarding viral loads (119). This might suggest a role for type II NKT cells in regulating host immune response rather than controlling viral replication.

The role of type I NKT cells in secondary bacterial infection post-influenza has also been examined. Using a model of post-IAV invasive secondary pneumococcal infection, type I NKT cells were shown to limit susceptibility to superinfection and lethal viral/bacterial synergism (112). In this setting, type I NKT cells act early after IAV infection by preventing tissue damages (through IL-22) (118). On the other hand, at the peak of bacterial susceptibility, type I NKT cells are in a state of hyporesponsiveness; an effect that depends on the immunosuppressive cytokine IL-10 (112). Interestingly, IL-10 blockade restored type I NKT cell activity and slightly increased resistance to bacterial superinfection (112). Of interest, α-GalCer instillation tempered secondary bacterial infection with lower bacterial outgrowth and dissemination (194) suggesting that type I NKT cells could be instrumental in clinics to combat IAV infection and its complications.

#### MAIT Cells

Since they cannot generate MAIT cell Ags derived from the riboflavin pathway, viruses were intuitively thought to be unable to activate MAIT cells. Nevertheless, MAIT cells can be activated in the absence of TCR ligation by many cytokines (e.g., IL-1β, IL-2, IL-7, IL-12, IL-15, IL-18, IL-23, and type I IFN) (75, 195, 196) released by accessory cells through engagement of various pattern recognition receptors including toll-like receptors and/or nod-like receptors.

Pioneer experiments demonstrated that MAIT cells isolated from Vα19iTg mice do not respond to DCs infected with a broad set of viruses (69). However, as previously mentioned, MAIT cells from Tg mice differ from conventional ones in that they present a naive profile and are devoid of the key transcription factor PLZF (67). Since PLZF controls expression of many chemokine/cytokine receptors (197) including IL-7Rα and IL-18Rα on MAIT cells (39), this can simply explain the absence of response of MAIT cells from Tg mice to virus-induced activating cytokines. The putative functions of MAIT cells during experimental influenza infection are still ignored.

Human MAIT cells can respond *in vitro* to IAV by CD69 and granzyme B upregulation as well as IFN-γ secretion (68, 75). In these settings, purified or enriched MAIT cells were cultured with peripheral blood mononuclear cells and IAV-infected epithelial cells or IAV-exposed macrophages. Mechanistically, MAIT cell activation was dependent on IL-18 secretion by monocyte/macrophages and did not require functional MR1 molecule (68, 75). Notably, IAV replication was not required since ultraviolet-irradiated IAV were still able to activate MAIT cells (75). These data suggest a potential role for MAIT cells in host response during IAV infection.

Compared to healthy donors, the frequency of circulating MAIT cells was decreased in a small cohort of 16 patients hospitalized for severe pneumonia due to infection with the Asian lineage avian IAV (H7N9) (68). Interestingly, individuals who recovered from pneumonia had higher levels of circulating MAIT cells compared with those who succumbed (68). Another clinical study confirmed the reduced MAIT cell frequencies in patients with acute IAV infection (75). This decrease was even more pronounced in critically ill patients admitted in intensive care unit compared to patients with mild symptoms (75).

### Future Directions

Natural killer T cells and MAIT cells participate in host response to numerous respiratory pathogens. These cells might have some overlapping functions during lung infections. To better elucidate their differential contribution, integrated analysis of activation dynamics and functions of NKT cells and MAIT cells in experimental models as well as in patients with severe lung infection should be encouraged. In addition to the models discussed above, NKT cells have been reported to play important role *in vivo* in many other areas of lung infections including respiratory syncytial virus, *P. aeruginosa, Legionella pneumophilia, Chlamydia* spp. as well as the fungi *Cryptococcus neoformans* and *Aspergillus fumigates* [for a review, see Ref. (198)]. Thus, it would be worth to examine the activation status and putative functions of MAIT cells in these models.

Regarding the limitations enumerated previously, the conclusions drawn from the phenotype observed in *J*α*18*^−/−^, *Cd1d*^−/−^, *Mr1*^−/−^, *V*α*14i* Tg, and *V*α*19i* Tg mice should be taken with caution. Therefore, reconstitution experiments with organ-specific cells in these knock-out/transgenic mice appear mandatory to confirm previous findings. In addition, selective *in vivo* depletion of these populations by means of monoclonal Ab represents an alternative approach as recently reported for type I NKT cells (199). Additionally, since these cells are likely to regulate homeostasis and functions of each other, crossing NKT cell- and MAIT cell-deficient mice will certainly provide new interesting tools to determine the specific or redundant functions of these cells.

Although the evolutionary conservation of CD1d and MR1 across mammals renders rodent models of pulmonary infections relevant to study NKT cell and MAIT cell biology, the expected widespread of humanized mouse models will surely provide new insights on the development and regulation of the immune response during lung infections. In addition, these models will be likely to highlight the NKT cell and MAIT cell species’ differences in data generated from mouse and human studies.

The current therapeutical arsenal for clinicians to combat lung infections is almost exclusively based on the use of antimicrobial drugs and standardized daily management. However, fatal outcomes in patients with severe lung infection are often associated with dysregulated immune response. In this condition, innovative strategies of immune intervention targeting host factors should be proposed. Thus, according to their immunoregulatory functions, NKT cells and MAIT cells are well poised for cell-based therapy to fight lung infections and their complications.

## Therapeutic Opportunities and Concluding Remarks

The last century has witnessed incredible progresses in the control of infectious diseases, which significantly reduced the human death toll, especially in western countries. However, the incidence of lung infections has significantly increased over the last decades due to major changes in environment and lifestyle (leading to immune homeostasis dysregulation and increased contact with new pathogens). These events represent key societal and public health threats. In addition, booming in resistance to antimicrobial drugs also constitute a new challenge for clinicians. Thus, the development of new therapeutical options to combat lung infections and their complications is urgently needed.

Harnessing the biology of CD1- and MR1-restricted cells is currently highly regarded to fight cancer (200, 201). By contrast, exploiting NKT cell and MAIT cell in clinical trials for lung infections is still in its infancy. Yet, targeting these cells offers several advantages that may lead to improve current management of lung infections. First, NKT cells and MAIT cells are restricted by quasi-monomorphic Ag-presenting molecules. Thus, they have the potential to be manipulated by “universal” ligands rendering most of patients eligible for NKT/MAIT cell-based therapies. Second, engineering of polarizing Ags (only developed for NKT cells so far) allows considering the generation of tailored-made responses according to the immune profile of the patients. Last, experimental and clinical studies indicated that NKT cell-based therapies are safe and feasible (202). Thus, many strategies might be tested to harness CD1d- and MR1-restricted T cell functions during lung infections.

The adjuvant properties of NKT cells and MAIT cells could be exploited in the design of more efficient vaccines. The replacement of conventional adjuvants by NKT/MAIT cell Ags (α-GalCer and its analogs, ribloflavin metabolites) in classical vaccines could be used to optimize the magnitude and duration of the adaptive immune. Through the ability of NKT cells and MAIT cells to subsequently activate/mature accessory cells including DCs, this strategy is likely to improve the development of the memory response.

In addition, these Ags might also generate short-term and organ-specific immune responses with positive outcomes (8). As clinical studies predominantly reported decreased levels of circulating NKT cells and MAIT cells in patients with severe pneumonia and associated with reduced prognosis, the proliferative activity (*in vivo* or *ex vivo*) of these Ags might help to the replenishment of the NKT/MAIT cell pool. Of note, the highly unstable nature of MAIT cell Ags precludes its use in expansion protocols and, therefore, represents a major limitation in these strategies. However, recent efforts in generating more stable forms of MAIT cell Ags (203, 204) should help to circumvent this issue in the near future. Despite being more laborious, the expansion of MAIT cells can also be achieved through reprogramming to pluripotent stem cells and subsequent redifferentiation using the stromal cell line OP9/DL1 (205).

The effects of combining NKT/MAIT cell Ags with antimicrobial drugs should also be encouraged. By reducing the concentrations of antibiotics or antiviral drugs used in conventional therapy for a similar antimicrobial activity, this strategy could help to lessen the development of drug resistance.

Chimeric Ag receptor (CAR) T cell therapy using conventional T cells has recently emerged as a promising approach in clinics (206). However, the high polymorphism in HLA system limits the development of CAR T cell therapy due to potential appearance of side effects such as graft-versus-host disease. Since many bacteria express glycolipid Ags for NKT cells, the recent generation of CAR-expressing NKT cells (207, 208) offers an interesting and highly innovative approach in the context of antibacterial therapies. Similar approaches could be developed in the future with CAR-expressing MAIT cells.

Dealing with their hyporesponsiveness during lung infections represents another food for thought for NKT/MAIT cell-based therapy. Increased expression of the checkpoint inhibitor PD-1 on NKT cells and MAIT cells in patients with TB has been associated with reduced capacity to produce antimycobacterial cytokines (136, 137, 147). Blockade of PD-1 expression has recently emerged as a potential strategy for infectious diseases including lung infection and sepsis (209–212). Thus, the possibility that the protective activity of anti-PD-1 treatments might partially rely on the restoration of NKT cell and MAIT cell functions should be considered. Treatment with α-GalCer has also been shown to lead to NKT cell hyporesponsiveness due to TCR internalization (213) and/or induction of PD-1 expression (214). New formulations and a better delivery of α-GalCer to potent Ag-presenting cells (such as DCs) by means of dedicated nanovectors can prevent the development of NKT cell anergy (215–218). These strategies might optimize NKT cell-based therapies to combat respiratory infections.

Natural killer T cells and MAIT cells have become the focus of intense investigation in the last decade. It is now clear that harnessing the biology of these cells has the potential to offer innovative therapeutic approaches in a medical field in which clinicians require a new and efficient arsenal to treat infections and their complications.

## Author Contributions

All authors listed have made a substantial, direct, and intellectual contribution to the work and approved it for publication.

## Conflict of Interest Statement

The authors declare that the research was conducted in the absence of any commercial or financial relationships that could be construed as a potential conflict of interest.

## Abbreviations

TCR, T cell receptor; MHC, major histocompatibility complex; Ag, antigen; NKT, natural killer T; MAIT, mucosal-associated invariant T; GSL, glycosphingolipids; α-GalCer, α-galactosylceramide; PLZF, pro-myelocytic leukemia zinc finger; Tg, transgenic; DC, dendritic cell; BCG, bacillus Calmette–Guérin; IAV, influenza A virus; Ab, antibody; WT, wild-type; CAR, chimeric Ag receptor.
